# Pharmaceutical care in Chinese public tertiary hospitals: findings from the 4th National Healthcare Improvement Initiative Survey

**DOI:** 10.1186/s12960-020-00473-z

**Published:** 2020-04-28

**Authors:** Mingshuang Li, Man Cao, Jing Sun, Yu Jiang, Yuanli Liu

**Affiliations:** School of Public Health, Chinese Academy of Medical Sciences and Peking Union Medical College, 5 Dongdansantiao, Dongcheng, Beijing, 100730 China

**Keywords:** Pharmaceutical care, Pharmacist, Survey, Public tertiary hospital, China

## Abstract

**Background:**

Pharmaceutical care has been playing an increasingly critical role in Chinese hospitals. However, evidence about the most recent development of pharmaceutical care in China is limited. This study analyzed the current situation of pharmaceutical care and the capacities of pharmacists in Chinese public tertiary hospitals.

**Methods:**

All on-duty pharmacists of 143 public tertiary hospitals responded to the Likert-5 pre-set online questionnaire about their pharmaceutical care capacities in eight aspects, and their respective hospitals valued pharmaceutical care in clinical practice from March 18 to 31, 2019. This study measured the appraisals of the responding pharmacists as positive (“strongly agree” and “agree”) or negative (“neither agree nor disagree,” “strongly disagree,” and “disagree”) results. The study performed a descriptive analysis of the responding pharmacists and unconditional multivariate binary logistic regression analysis to predict the influencing factors of the pharmacists’ appraisals of pharmaceutical care. The dependent variable was transformed into binary categories and assigned 1 = positive response and 0 = negative response. The independent variables included the identifications of sample hospitals and the characteristics of the responding pharmacists.

**Results:**

The survey retrieved 10 815 valid responded questionnaires. 74.5%, 67.5%, and 65.0% of the responding pharmacists made a positive self-appraisal of their abilities of communication with patients and doctors, reviewing prescriptions, and communication with particular patients, respectively. 65.5% had a positive appraisal of their respective hospitals to conduct active monitoring of the clinical use of new medicines, and 68.9% admitted that their respective hospitals valued the clinical pharmaceutical care. The doctor’s degree and senior academic rank of the responding pharmacists, as well as maternal and child hospitals, were predictors of higher appraisals of the responding pharmacists about their pharmaceutical care capacities, and their respective hospitals valued pharmaceutical care (all ORs > 1.5, *P* < 0.05).

**Conclusions:**

The study suggested that there is a need to strengthen the pharmacy education, training, and staffing of pharmacists with improved professional skills to offer value-added specialized pharmaceutical care in Chinese public hospitals. Patient-centered and inter-disciplinary interactions in medical practice should be promoted. There is also a need for public hospitals to provide a platform for the achievement of the professional values of high-quality pharmacists at different carrier development stages.

## Introduction

Professor Robert L Mikeal firstly raised the concept of pharmaceutical care in 1975. Hepler and Strand proposed the most widely accepted international concept of pharmaceutical care in 1990, which referred the care to provide pharmaco-therapy to patients to achieve specific health outcomes that expect to improve patient’s quality of life [1]. In 2013, the Pharmaceutical Care Network Europe reached a new consensus on the definition of pharmaceutical care, which was the pharmacist’s contribution to the care of individuals to optimize medicines use and to improve health outcomes [2]. Modern pharmaceutical care has been evolving from supply of medicines to clinical practice that covers the entire process of medical care, including the selection of medicines, design and adjustment of treatment regimens, monitor the efficacy of medication, prevention, monitor and treatment of adverse drug reactions, and guide prevention and medication treatment. Moreover, the core of pharmaceutical care has been shifting from “medicines” to “patients.” Pharmacists have been increasingly expected to use their professional knowledge and skills to help patients for achieving satisfactory health outcomes and reducing medical expenditures.

The development of pharmaceutical care in China lagged behind the Western world. In 2002, the Chinese Ministry of Health firstly proposed to establish a clinical pharmacist system in hospitals, required pharmacists to participate in clinical practice [3], and called for a patient-centered comprehensive clinical practice model [4]. In 2007, some pilot programs marked the initiation of the development of the clinical pharmacist system in China [5]. Subsequently, all public hospitals are required to set up a technical team of clinical pharmacists [6].

Pharmaceutical care in Chinese hospitals has been evolving from the simple provision of medicines to patient-centered care focusing on interactions between patients and medications [7–9]. Pharmacists have been playing a more critical role in hospital clinical practice [10–13]. However, except for some preliminary and theoretical review of the pharmaceutical care in the Chinese hospital in the early 2010s, little is known about the most recent development of the pharmaceutical care and the capacities of the pharmacists in Chinese public hospitals. Unlike the United States of America’s National Clinical Pharmacy Services Surveys in Hospitals conducted every 3 years, there is yet a national review of clinical pharmacy services and pharmacist staffing in China. The 4th National Healthcare Improvement Initiative Survey in 2019 brought an opportunity to the study team to incorporate a national pharmacist survey into the overall survey, conducted in the public tertiary hospitals across the country. It is by far the first nationwide pharmacist survey conducted in all types of public tertiary hospitals across 31 provinces of China. This paper analyzed the key finding from the national pharmacist survey and showed the capacities of hospital pharmacists in delivering pharmaceutical care and if their respective hospitals valued the care from the perspective of the pharmacist.

## Methods

### Population and setting

One hundred forty-three public tertiary hospitals across China carried out the national pharmacist survey from March 18 to 31, 2019. In each of the 31 provinces, the national survey selected one provincial general hospital, one provincial traditional Chinese medicine (TCM) hospital, and one maternal and child health (MCH) hospital. Besides, 50 National Health Commission (NHC)- and National Administration of Traditional Chinese Medicine (NATCM)-affiliated hospitals (including 28 general hospitals, seven TCM hospitals, and 15 specialist hospitals) were also selected. All of the selected hospitals are public tertiary hospitals. All pharmacists of the sample hospitals on duty during the time of the national sure participated.

### Measurements

The survey used a Likert-5 scale to formulate the questionnaire to understand the practice and capacity of delivering pharmaceutical care from the perspective of the pharmacist. Essential contents of the questionnaire include (1) necessary information of the sample hospitals; (2) self-appraisal of the responding pharmacists about their pharmaceutical care capabilities, including the ability of acquiring medication information of patients promptly, reviewing prescriptions, and cooperating with doctors to select medicines, guidance of medication, telemedicine consultation, individualized medication, and communication with doctors and patients, especially particular patients; and (3) appraisal of the responding pharmacists about the pharmaceutical care in their respective hospitals, including carried out active monitor of clinical use of new medicines, and valued the pharmaceutical care in clinical practice. Pharmacists who responded to the pre-set online questionnaire were required to choose one of the following assessments: “strongly agree,” “agree,” “neither agree nor disagree,” “disagree,” or “strongly disagree” to each of the statement of the above-concerned issues.

### Statistical analysis

The study measured the appraisal of the responding pharmacists by calculating the percentage of the respondent frequency for each of the Likert-5 scales and compared the proportion of the positive responses (“strongly agree” and “agree”) versus negative responses (“neither agree nor disagree,” “strongly disagree,” and “disagree”) for each of the statement. This study used the SPSS 25.0 software to perform the following tasks: (1) descriptive analysis of the demographic, social, and geographic characteristics of the responding pharmacists; (2) calculation of he respondent frequency for each of the Likert-5 scales and the proportion of the positive versus negative response for each of the statements; and (3) univariate analyses of the differences of the appraisals among different groups of the responding pharmacists with *χ*^2^ test; and (4) unconditional multivariate binary logistic regression analysis to predict the influencing factors of the pharmacists’ appraisals of pharmaceutical care. The dependent variable was transformed into binary categories and assigned 1 = positive response and 0 = negative response. The independent variables included the identifications of sample hospitals and the characteristics of the responding pharmacists. The significant level was set as *P* < 0.05.

## Results

### Characteristics of the responding pharmacists

A total of 10 815 pharmacists responded to the national pharmacist survey, among which, 68.2% were females, more than twice the number of the males. The mean age of the responding pharmacists was 36.2 (min = 20, max = 76, median = 34). Their average length of work experience was 13.3 years (min = 1 year, max = 54 years, median = 10 years). The proportion of pharmacists with senior, middle, and junior academic rank was 12.35%, 38.0%, and 49.7%, respectively. The proportion of pharmacists with the highest degree of doctor, master, bachelor, or lower level was 3.5%, 20.5%, 62.4%, and 13.6%, respectively. Among the 143 sample hospitals, the average proportion of the number of pharmacists to the total number of health professionals was 4.8% (min = 0.9%, max = 14.7%, median = 3.9%). The hospitals with a proportion of 8% or above, 5–8%, and < 5% of the number of pharmacists to the total number of health professionals accounted for 14.0%, 18.2%, and 67.8% of the total 143 sample hospitals, respectively.

As presented in Additional file 1, there were significant differences in the distribution of age, length of work experience, academic rank, and the highest degree among the responding pharmacists in the eastern, central, and western regions, from the NHC- or NATCM-affiliated hospitals and local hospitals and from the general, TCM, MCH, and specialist hospitals (*P* < 0.01). The differences in gender distribution were significant among the responding pharmacists in different areas and different types of hospitals (*P* < 0.01).

### Self-appraisal of the responding pharmacists about their pharmaceutical care capabilities

74.5%, 67.5%, and 65.0% of the responding pharmacists made a positive self-appraisal of their abilities of communication with patients and doctors, reviewing prescriptions, and communication with particular patients, respectively. 17.0%, 15.8%, and 12.8% of the responding pharmacists made a negative self-appraisal of their abilities to conduct individualized medication, telemedicine consultation, and cooperation with doctors to select medicines, respectively.

The logistic regression model included the characteristics of the responding pharmacists (gender, age, academic rank, length of work experience, and highest degree) and their respective hospitals (region, affiliation, type, and proportion of the number of pharmacists to the total number of health professionals) with the backward regression. The study set the exclusion threshold at 0.10. The Hosmer-Lemeshow goodness-of-fit test was adopted to secure that the fitting effect of each model was good (*P* > 0.05). Table 1 shows the results of the regressions. The likelihoods of theresponding pharmacists with a higher degree, especially those with a doctor's degree to give positive self-appraisals about their pharmaceutical care capacities were much higher than those with a bachelor's degree or lower (OR = 1.56–2.80, *P* < 0.01). The likelihoods of the responding pharmacists with the senior academic rank to give positive self-appraisals about their abilities to offer medication guidance and individualized medication were much higher than those with the junior academic rank (OR = 1.64/1.69, *P* < 0.01), respectively. The likelihoods of the responding pharmacists from the MCH (OR = 2.17, *P* > 0.01) & TCM (OR = 1.76, *P* < 0.03) hospitals to give positive self-appraisals about their abilities to acquire medication information of patients promptly were much higher than those from the specialist hosptials. The likelihoods of the responding pharmacists fromthe MCH hospitals to give positive self-appraisals about their abilities of providing medication guide to individual patients, telemedicine consultation, and communication with particular patients were much higher than those from the speciaist hospitals (OR = 1.75–1.95, *P* &lt; 0.05).

**Table 1:** Multivariate logistic regression analysis results of the self-appraisals of the responding pharmacists about their capacities to provide pharmaceutical care

Capacities to provide pharmaceutical care	Coefficient	Standard error	Wald	*P*	Odds ratio	95% CI	
						Lower	Upper
1. The ability to acquire medication information of patients promptly							
Region (ref. western)							
Eastern	− 0.25	0.05	23.22	< 0.01	0.78	0.71	0.86
Middle	− 0.16	0.06	8.13	< 0.01	0.85	0.76	0.95
Type of hospital (ref. specialist hospital)							
**TCM hospital**	**0.57**	**0.27**	**4.52**	**0.03**	**1.76**	**1.05**	**2.97**
**MCH hospital**	**0.77**	**0.27**	**8.30**	**< 0.01**	**2.17**	**1.28**	**3.67**
Male (ref. female)	0.11	0.04	6.63	0.01	1.12	1.03	1.21
Length of work experience (ref. > 30 years)							
20–30 years	− 0.26	0.09	8.31	< 0.01	0.77	0.64	0.92
Academic rank (ref. junior)							
Middle	− 0.18	0.05	13.34	< 0.01	0.83	0.75	0.92
Highest degree (ref. below bachelor’s degree)							
**Doctor’s degree**	**0.78**	**0.14**	**32.28**	**< 0.01**	**2.18**	**1.67**	**2.86**
Master’s degree	0.35	0.08	17.76	< 0.01	1.42	1.21	1.67
2. The ability to review prescriptions							
Region (ref. western)							
Eastern	− 0.21	0.05	16.27	< 0.01	0.81	0.73	0.90
Middle	− 0.39	0.06	43.64	< 0.01	0.68	0.60	0.76
Length of work experience (ref. > 30 years)							
< 10 years	− 0.31	0.11	8.16	< 0.01	0.74	0.60	0.91
10–20 years	− 0.22	0.10	4.48	0.03	0.81	0.66	0.98
20–30 years	− 0.23	0.10	5.65	0.02	0.79	0.66	0.96
Academic rank (ref. junior)							
Senior	− 0.23	0.09	6.69	0.01	0.79	0.67	0.95
Middle	− 0.18	0.05	12.02	< 0.01	0.83	0.75	0.92
Highest degree (ref. below bachelor’s degree)							
**Doctor’s degree**	**0.44**	**0.14**	**10.20**	**< 0.01**	**1.56**	**1.19**	**2.04**
Bachelor’s degree	0.32	0.07	21.77	< 0.01	1.37	1.20	1.57
3. The ability to cooperate with doctors to select medicines							
Region (ref. western)							
Eastern	− 0.16	0.05	10.56	< 0.01	0.85	0.78	0.94
Middle	− 0.15	0.06	6.87	0.01	0.86	0.77	0.96
Male (ref. female)	0.16	0.04	14.15	< 0.01	1.17	1.08	1.27
Length of work experience (ref. > 30 years)							
< 10 years	− 0.35	0.10	12.37	< 0.01	0.70	0.58	0.86
10–20 years	− 0.32	0.10	11.40	< 0.01	0.72	0.60	0.87
20–30 years	− 0.38	0.09	17.55	< 0.01	0.68	0.57	0.82
Academic rank (ref. junior)							
Senior	0.28	0.08	11.30	< 0.01	1.33	1.13	1.57
Highest degree (ref. below bachelor’s degree)							
**Doctor’s degree**	**0.89**	**0.14**	**43.41**	**< 0.01**	**2.43**	**1.87**	**3.17**
Master’s degree	0.42	0.08	25.60	< 0.01	1.52	1.29	1.78
4. The ability to provide medication guidance							
Region (ref. western)							
Eastern	− 0.15	0.05	8.95	< 0.01	0.86	0.78	0.95
Type of hospital (ref. other specialist hospitals)							
**MCH hospital**	**0.65**	**0.27**	**5.78**	**0.02**	**1.92**	**1.13**	**3.28**
Male (ref. female)	0.25	0.04	34.68	< 0.01	1.28	1.18	1.40
Length of work experience (ref. > 30 years)							
< 10 years	− 0.38	0.10	14.02	< 0.01	0.69	0.56	0.84
10–20 years	− 0.25	0.10	6.89	0.01	0.78	0.64	0.94
20–30 years	− 0.27	0.09	8.47	< 0.01	0.77	0.64	0.92
Academic rank (ref. junior)							
**Senior**	**0.49**	**0.09**	**33.82**	**< 0.01**	**1.64**	**1.39**	**1.93**
Highest degree (ref. below bachelor’s degree)							
**Doctor’s degree**	**0.54**	**0.13**	**16.76**	**< 0.01**	**1.72**	**1.33**	**2.23**
5. The ability of telemedicine consultation							
Region (ref. western)							
Eastern	− 0.12	0.05	5.33	0.02	0.89	0.80	0.98
Affiliation of hospital (ref. local hospital)							
NHC-affiliated hospital	0.16	0.05	10.57	< 0.01	1.18	1.07	1.30
Type of hospital (ref. specialist hospital)							
**MCH hospital**	**0.56**	**0.28**	**4.05**	**0.04**	**1.75**	**1.02**	**3.00**
Male (ref. female)	0.27	0.04	40.81	< 0.01	1.31	1.21	1.43
Academic rank (ref. junior)							
** Senior**	**0.35**	**0.09**	**15.86**	**< 0.01**	**1.42**	**1.20**	**1.69**
Highest degree (ref. below bachelor’s degree)							
**Doctor’s degree**	**0.96**	**0.14**	**50.89**	**< 0.01**	**2.62**	**2.01**	**3.41**
**Master’s degree**	**0.47**	**0.08**	**32.13**	**< 0.01**	**1.61**	**1.36**	**1.89**
Bachelor’s degree	0.14	0.07	4.26	0.04	1.15	1.01	1.31
6. The ability of individualized medication							
Region (ref. western)							
Eastern	− 0.18	0.05	12.55	< 0.01	0.84	0.76	0.93
Male (ref. female)	0.28	0.04	42.96	< 0.01	1.33	1.22	1.45
Length of work experience (ref. > 30 years)							
< 10 years	− 0.22	0.10	4.60	0.03	0.80	0.66	0.98
10–20 years	− 0.23	0.10	5.56	0.02	0.80	0.66	0.96
20–30 years	− 0.37	0.09	16.35	< 0.01	0.69	0.58	0.83
Academic rank (ref. junior)							
** Senior**	**0.53**	**0.09**	**37.81**	**< 0.01**	**1.69**	**1.43**	**2.00**
Highest degree (ref. below bachelor’s degree)							
**Doctor’s degree**	**1.03**	**0.14**	**58.04**	**< 0.01**	**2.80**	**2.15**	**3.64**
**Master’s degree**	**0.57**	**0.08**	**45.28**	**< 0.01**	**1.77**	**1.50**	**2.08**
7. The ability of communication with particular patients							
Region (ref. western)							
Eastern	− 0.25	0.05	23.80	< 0.01	0.78	0.71	0.86
Middle	− 0.17	0.06	8.77	< 0.01	0.84	0.75	0.94
Type of hospital (ref. specialist hospital)							
**MCH hospital**	**0.67**	**0.26**	**6.57**	**0.01**	**1.95**	**1.17**	**3.25**
Length of work experience (ref. > 30 years)							
< 10 years	− 0.26	0.11	5.87	0.02	0.77	0.63	0.95
Highest degree (ref. below bachelor’s degree)							
**Doctor’s degree**	**0.62**	**0.14**	**18.68**	**< 0.01**	**1.85**	**1.40**	**2.45**
Master’s degree	0.19	0.09	5.16	0.02	1.21	1.03	1.44
8. The ability of communication with doctors and patients							
Region (ref. western)							
Eastern	− 0.32	0.06	32.14	< 0.01	0.72	0.65	0.81
Middle	− 0.28	0.07	19.10	< 0.01	0.75	0.66	0.86
Length of work experience (ref. > 30 years)							
< 10 years	− 0.53	0.12	19.05	< 0.01	0.59	0.47	0.75
10–20 years	− 0.32	0.12	7.68	0.01	0.72	0.58	0.91
Highest degree (ref. below bachelor’s degree)							
**Doctor’s degree**	**0.64**	**0.16**	**16.40**	**< 0.01**	**1.90**	**1.39**	**2.59**
Master’s degree	0.23	0.09	6.03	0.01	1.25	1.05	1.50
Bachelor’s degree	0.24	0.07	10.66	< 0.01	1.27	1.10	1.46

Bold signifies protective factors

*MCH* maternal and child health, *TCM* traditional Chinese medicine

### Appraisal of the responding pharmacists about their respective hospitals valued pharmaceutical care in clinical practice

65.5% of the responding pharmacists had a positive appraisal of their respective hospitals to conduct active monitoring of the clinical use of new medicines, and 68.9% admitted that their respective hospitals valued the clinical pharmaceutical care.

The study conducted the similar multivariate logistic regression analyses to predict the factors that affect the appraisal results of the responding pharmacists if their respective hospitals conducted active monitoring of the clinical use of new medicines, and valued the pharmaceutical care. Table 2 shows the results of the regressions. The likelihoods of the responding pharmacists in NHC-affiliated hospitals to give positive appraisals about their respective hospitals to conduct active monitoring of the clinical use of new medicines, and to value pharmaceutical care in clinical practice were much higher than those form the specialist hospitals (OR = 1.44/1.43, *P* < 0.01). The likelihood of the responding pharmacists with a doctor's degree to give positive appraisals to their respective hostpitals tovalue the monitoging of clinical use of new medicines was much higher than those with a lower than bachelor's degree (OR = 1.47, *P* = 0.01).

**Table 2:** Multivariate logistic regression analysis results of the appraisal of the responding pharmacists about whether their respective hospitals valued the pharmaceutical care in clinical practice

Variables	Coefficients	Standard error	Wald	*P*	Odds ratio	95% CI	
						Lower	Upper
1. The respective hospital conducted active monitoring of the clinical use of new medicines							
Region (ref. western)							
Eastern	− 0.27	0.05	25.62	< 0.01	0.76	0.69	0.85
Affiliation of hospital (ref. local hospital)							
**NHC-affiliated hospital**	**0.36**	**0.05**	**47.89**	**< 0.01**	**1.44**	**1.30**	**1.59**
NATCM-affiliated hospital	− 0.33	0.10	11.47	< 0.01	0.72	0.59	0.87
Length of work experience (ref. >30 years)							
< 10 years	− 0.29	0.11	7.73	0.01	0.75	0.61	0.92
10–20 years	− 0.27	0.10	6.98	0.01	0.77	0.63	0.93
20–30 years	− 0.30	0.10	10.00	< 0.01	0.74	0.61	0.89
Academic rank (ref. junior)							
Senior	− 0.32	0.09	13.33	< 0.01	0.73	0.61	0.86
Middle	− 0.31	0.05	35.06	< 0.01	0.73	0.66	0.81
Highest degree (ref. below bachelor’s degree)							
**Doctor’s degree**	**0.39**	**0.14**	**7.42**	**0.01**	**1.47**	**1.12**	**1.95**
2. The respective hospital valued pharmaceutical care in clinical practice							
Region (ref. western)							
Eastern	− 0.32	0.06	32.24	< 0.01	0.73	0.65	0.81
Affiliation of hospital (ref. local hospital)							
** NHC-affiliated hospital**	**0.36**	**0.05**	**44.24**	**< 0.01**	**1.43**	**1.29**	**1.59**
Male (ref. female)	− 0.12	0.05	7.22	0.01	0.89	0.81	0.97
Length of work experience (ref. > 30 years)							
< 10 years	− 0.48	0.11	18.85	< 0.01	0.62	0.50	0.77
10–20 years	− 0.49	0.11	21.41	< 0.01	0.61	0.50	0.75
20–30 years	− 0.39	0.10	14.66	< 0.01	0.68	0.56	0.83
Academic rank (ref. junior)							
Senior	− 0.33	0.09	13.40	< 0.01	0.72	0.60	0.86
Middle	− 0.35	0.05	41.70	< 0.01	0.71	0.64	0.79
The proportion of the number of pharmacists to the total number of health professionals (ref. > 10%)							
< 5%	0.17	0.06	7.53	0.01	1.18	1.05	1.33
5–8%	0.21	0.07	8.99	< 0.01	1.24	1.08	1.42

Bold signifies protective factors

*NHC* National Health Commission, *NATCM* National Administration of Traditional Chinese Medicine

## Discussion

The study found that, among the 143 public tertiary hospitals, the average proportion of the number of pharmacists to the total number of health professionals was 4.8%, which was consistent with the national average of 4.5% as reported by the China Health Statistics Yearbook in 2017 [14]. According to the “Regulations on the Administration of Pharmaceutical Care in Hospitals” implemented in 2011, public hospitals should have at least 8% of the total number of health professionals specialized in pharmaceutical care [6]. Both the national average in 2017 and the results of this survey conducted in 2019 are far below the target set by the government in 2011. Xi et al. [15] surveyed 292 Chinese public tertiary hospitals in 2017 and found that the average number of pharmacists per 100 beds was 5.6, and the average number of clinical pharmacists was 0.43 per 100 beds, which was only about half of the pharmacists (9.77 per 100 beds) and less than 20% of clinical pharmacists (2.42 per 100 beds) in the United States of America in 2007 [16, 17].

In particular, the sample hospitals of this survey are the top public tertiary hospitals across the country, which more powerfully implies that pharmacists in Chinese public hospitals are understaconducted in 2019 fing. The reasons behind the understaffing of pharmacists are multiple. Apart from that, clinical pharmaceutical care in hospitals are still in the initial development stage in China; most hospitals yet fully recognized the value of pharmaceutical care in clinical practice and established patient-centered pharmaceutical care. The understaffing of pharmacists may also be associated with the “zero mark-up policy on medicines” being implemented in the public hospitals in China. When the mark-up on medicines was removed; the consultation fee and pricing for medical services were not timely adjusted to reflect the value of the care.

Further to this, the insufficient government subsidies and the overall revenue generated by the public hospitals were not enough to keep the routine operation, which brought economic pressure to many public hospitals. Public hospitals regard pharmacists as non-revenue generators and control or even reduce the number of pharmacists. Some public hospitals even outsourced pharmacy services and shifted the function of pharmacy management to the third party commercial companies [18–21]. Public hospitals generally do not value the professionalism of pharmaceutical care with a profit-driven philosophy under such circumstances [22].

The study also found that nearly 80% of the responding pharmacists had a bachelor’s or lower degree, and half of them had a junior or lower academic rank. It means that Chinese public hospitals need to strengthen the professionalism of pharmaceutical care. Compared with the developed settings in the United States of America, almost all active pharmacists in clinical practice have a pharmaceutical doctoral education background [23]. It takes at least 6 years to complete a doctoral education in pharmacy in the United States of America. The strict training system in quite a large number of School of Pharmacy in the United States of America has formulated a large number of pharmacist professionals with excellent knowledge and skills [24]. While in China, there is only a very few numbers of School of Pharmacy offer PharmD degree education, and most of the curriculums follow the traditional training model, focused very much on chemistry, which leads to a dilemma of lack of clinical thinking and insufficient communication skills and practical capability [25]. Many pharmacists are yet qualified to provide professional medication guidance in clinical practice [26]. There is a need to improve the curriculum of pharmacy and pay more attention to practice and inter-disciplinary education [27].

Compared with the core set of services provided in the hospitals in the United States of America (drug information, adverse drug reaction management, drug protocol management, medical rounds, and admission drug histories) [28], the critical functions of the Department of Pharmacy in most of the Chinese hospitals are still procurement, warehousing, and dispensing dominated. The function of pharmacists in offering guidance of medications to patients is limited. Clinical pharmacist is still a new professional post in Chinese hospitals; the role and function of clinical pharmacist need to be further defined [29]. The limited academic education and knowledge structure of the pharmacists, as well as understaffing in Chinese hospitals, also undermine the development of clinical pharmaceutical care.

The national pharmacist survey demonstrated that most of the responding pharmacists could communicate well with doctors, nurses, and patients; appropriately review prescriptions; and acquire the medication information of patients promptly. However, nearly 20% of the responding pharmacists were not satisfied with their abilities to provide individualized medication therapy, telemedicine consultation, and cooperate with doctors to select medicines, which were all within the scope of the core set of services provided in the hospitals in the United States of America [28]. The reasons behind such a gap are not only associated with the different education system of pharmacy in the two countries, but also the different healthcare service delivery modes. Chinese hospitals yet developed the multi-disciplinary treatment (MDT) model and integrated care as developed in US hospitals, where collaborative pharmacotherapy management project enables the pharmacists and physicians to collaborate in making clinical therapeutic decisions and managing the drug treatment of patients [29]. Chinese people have developed the deep-rooted belief of “seeking a doctor when sick” but ignore the role of a pharmacist.

There has been an imbalance of economic development in different regions of China. The eastern region is more developed than the west, while the western region is relatively backward. However, the national pharmacist survey showed that the appraisal results about the pharmaceutical care capacities of the responding pharmacists in the eastern region were all significantly lower than that of those in the western region. It might imply that the level of economic development is not an obstacle to the development of clinical pharmaceutical care, while the most critical factor might lie in the degree of attention. This finding is different from the results of an evaluation of hospital demographics and clinical pharmacist staffing/occupied bed in the United States of America. The US survey found that there were statistically significant associations between demographic variables and the clinical pharmacist staffing/occupied bed, the more accessible educational and training opportunities, the higher clinical pharmacist staffing, and more benefits of clinical pharmacy services on cost or patient care outcomes [17].

The results of the multivariate regression analyses showed that high academic rank and high degree were critical for the self-appraisal about the pharmaceutical care abilities of the responding pharmacists, as well as their respective hospitals to value the pharmaceutical care. The results further suggest that Chinese public hospitals need high-quality pharmacy talents to carry out value-added pharmaceutical care. More responding pharmacists at their middle professional carrier development stage were dissatisfied with their pharmaceutical care capacities compared with those who were at junior and senior stages, which indicates that pharmacists are subjectively willing to be actively involved in clinical pharmaceutical care. It also reminds the administrative departments to pay attention to the needs of the pharmacists for their carrier development, and to promote the professionalism of clinical pharmaceutical care, thus to strengthen the talent pool development.

The results of the multivariate regression analyses also showed that the NHC-affiliated hospitals might more value the clinical pharmaceutical care than the local hospitals according to the appraisal results of the responding pharmacists. Most NHC-affiliated hospitals are major teaching hospitals. This finding is in line with the results of Bond et al.’s evaluation of hospital demographics and clinical pharmacist staffing/occupied bed in the United States of America. The US evaluation found that hospitals affiliated with pharmacy teaching programs had significantly more clinical pharmacy services than those not so affiliated, and major teaching hospitals had substantially more clinical pharmacy services than those that were not major teaching hospitals [17]. This may suggest that local hospitals should further strengthen pharmaceutical care. As the number of local hospitals is large, the improvement of pharmaceutical care in local hospitals will benefit more patients.

Compared with the responding pharmacists with junior academic rank, those with middle and senior academic ranks had lower appraisals about active monitoring of clinical use of new medicines and value of the pharmaceutical care in clinical practice in their respective hospitals. It might be due to that pharmacists with junior and lower academic ranks are the main force of routine work and have a higher level of involvement in pharmaceutical care. It might also be associated with the higher expectations of the pharmacists with middle and senior academic rank. In short, the study calls for Chinese the public tertiary hospitals to strengthen the staffing of pharmaceutical professionals with high qualification and to improve their professional skills of offering value-added specialized pharmaceutical care. At the same time, public hospitals should further transform the pharmaceutical care into a “patient-centered” model and provide a platform for the achievement of the professional value of high-quality pharmacists at different stages of carrier development.

## Conclusions

The study suggested that there is a need to strengthen the pharmacy education, training, and staffing of pharmacists with improved professional skills to offer value-added specialized pharmaceutical care in Chinese public hospitals. Patient-centered and inter-disciplinary interactions in medical practice should be promoted. There is also a need for the public hospitals to provide a platform for the achievement of the professional values of high-quality pharmacists at different carrier development stages.

## Supplementary information

**Additional file 1.** Characteristics of the responding pharmacists from different regions, affiliated hospitals, and types of hospitals.

## Data Availability

Data supporting the results reported in the article are available from the corresponding author upon reasonable request.

## Abbreviations

MCH: Maternal and child health
MDT: Multi-disciplinary treatment
NATCM: National Administration of Traditional Chinese Medicine
NHC: National Health Commission
TCM: Traditional Chinese medicine
