# Supplementary material for: Pharmaceutical care in Chinese public tertiary hospitals: findings from the 4th National Healthcare Improvement Initiative Survey
Source: Hum Resour Health. 2020 Apr 28;18:31. doi: 10.1186/s12960-020-00473-z (PMC7189700; doi:10.1186/s12960-020-00473-z)
Supplement: Supplementary file 1 — Additional file 1. Characteristics of the responding pharmacists from different regions, affiliated hospitals, and types of hospitals. [file 12960_2020_473_MOESM1_ESM.docx]

**Pharmaceutical Care in Chinese Public Tertiary Hospitals – Findings from the 4th National Healthcare Improvement Initiative Survey**

**Appendix 1 Characteristics of the responding pharmacists from different regions, affiliated hospitals, and types of hospitals**

| **Characteristics of the responding pharmacists** | **Total**  **Sample n(%)** | **Region** | | | | | **Affiliation** | | | | | **Type of hospital** | | | | | |
| --- | --- | --- | --- | --- | --- | --- | --- | --- | --- | --- | --- | --- | --- | --- | --- | --- | --- |
|  |  | **Eastern**  **n(%)** | **Central**  **n(%)** | **Western**  **n(%)** | ***χ^2^*** | ***P*** | **NHC**  **n(%)** | **NATCM n(%)** | **Local**  **n(%)** | ***χ^2^*** | ***P*** | **General**  **n(%)** | **Traditional Chinese medicine**  **n(%)** | **Maternal and child**  **n(%)** | **Specialist types**  **n(%)** | ***χ^2^*** | ***P*** |
| **Total** | **10 815** | 5 276 | 2 733 | 2 806 |  |  | 3 431 | 570 | 6 814 |  |  | 5 489 | 3 250 | 2 013 | 63 |  |  |
| **Gender** |  |  |  |  | **8.6** | **0.01** |  |  |  | 5.7 | 0.06 |  |  |  |  | **17.7** | **＜0.01** |
| Male | 3 440(31.8) | 1 625(30.8) | 929(34.0) | 886(31.6) |  |  | 1 065(31.0) | 161(28.2) | 2 214(32.5) |  |  | 1 720(31.3) | 1 114(34.3) | 592(29.4) | 14(22.2) |  |  |
| Female | 7 375(68.2) | 3 651(69.2) | 1 804(66.0) | 1 920(68.4) |  |  | 2 366(69.0) | 409(71.8) | 4 600(67.5) |  |  | 3 769(68.7) | 2 136(65.7) | 1 421(70.6) | 49(77.8) |  |  |
| **Age** |  |  |  |  | **61.1** | **<0.01** |  |  |  | **56.1** | **<0.01** |  |  |  |  | **50.5** | **<0.01** |
| <30 | 2 454(22.7) | 1 121(21.2) | 636(23.3) | 697(24.8) |  |  | 905(26.4) | 126(22.1) | 1 423(20.9) |  |  | 1 279(23.3) | 671(20.6) | 491(24.4) | 13(20.6) |  |  |
| 30-40 | 5 101(47.2) | 2 470(46.8) | 1 300(47.6) | 1 331(47.4) |  |  | 1 588(46.3) | 269(47.2) | 3 244(47.6) |  |  | 2 457(44.8) | 1 599(49.2) | 1 015(50.4) | 30(47.6) |  |  |
| 40-50 | 2 011(18.6) | 1 112(21.1) | 488(17.9) | 411(14.6) |  |  | 622(18.1) | 108(18.9) | 1 281(18.8) |  |  | 1 055(19.2) | 629(19.4) | 314(15.6) | 13(20.6) |  |  |
| >50 | 1 249(11.5) | 573(10.9) | 309(11.3) | 367(13.1) |  |  | 316(9.2) | 67(11.8) | 866(12.7) |  |  | 698(12.7) | 351(10.8) | 193(9.6) | 7(11.1) |  |  |
| **Length of work experience** |  |  |  |  | **143.9** | **<0.01** |  |  |  | **88.2** | **<0.01** |  |  |  |  | **44.6** | **<0.01** |
| <10 years | 5 733(53.0) | 2 524(47.8) | 1 591(58.2) | 1 618(57.7) |  |  | 1 936(56.4) | 245(43.0) | 3 552(52.1) |  |  | 2 863(52.2) | 1672(51.4) | 1 168(58.0) | 30(47.6) |  |  |
| 10-20 years | 2 533(23.4) | 1 447(27.4) | 525(19.2) | 561(20.0) |  |  | 803(23.4) | 196(34.4) | 1 534(22.5) |  |  | 1 239(22.6) | 817(25.1) | 462(23.0) | 15(23.8) |  |  |
| 20-30 years | 1 807(16.8) | 960(18.2) | 440(16.1) | 407(14.5) |  |  | 524(15.3) | 94(16.5) | 1 189(17.4) |  |  | 981(17.9) | 550(16.9) | 263(13.1) | 13(20.6) |  |  |
| >30 years | 742(6.8) | 345(6.5) | 177(6.5) | 220(7.8) |  |  | 168(4.9) | 35(6.1) | 539(7.9) |  |  | 406(7.4) | 211(6.5) | 120(6.0) | 5(7.9) |  |  |
| **Academic rank** |  |  |  |  | **91.8** | **<0.01** |  |  |  | **151.2** | **<0.01** |  |  |  |  | **24.1** | **＜0.01** |
| Junior and blew | 5 382(49.7) | 2 555(48.4) | 1 297(47.5) | 1 530(54.5) |  |  | 1 889(55.1) | 298(52.3) | 3 195(46.9) |  |  | 2 633(48.0) | 1652(50.8) | 1 071(53.2) | 26(41.3) |  |  |
| Middle | 4 112(38.0) | 2 172(41.2) | 1 059(38.7) | 881(31.4) |  |  | 1279(37.3) | 236(41.4) | 2 597(38.1) |  |  | 2 170(39.5) | 1184(36.4) | 728(36.2) | 30(47.6) |  |  |
| Senior | 1 321(12.3) | 549(10.4) | 377(13.8) | 395(14.1) |  |  | 263(7.7) | 36(6.3) | 1 022(15.0) |  |  | 686(12.5) | 414(12.7) | 214(10.6) | 7(11.1) |  |  |
| **Highest degree** | |  |  |  | **278.7** | **<0.01** |  |  |  | **219.8** | **<0.01** |  |  |  |  | **203.4** | **<0.01** |
| Below Bachelor degree | 1 466(13.6) | 817(15.5) | 244(8.9) | 405(14.4) |  |  | 454(13.2) | 113(19.8) | 899(13.2) |  |  | 702(12.8) | 516(15.9) | 242(12.0) | 6(9.5) |  |  |
| Bachelor degree | 6 753(62.4) | 3 436(65.1) | 1 535(56.2) | 1782(63.5) |  |  | 1 985(57.9) | 412(72.3) | 4 356(63.9) |  |  | 3 262(59.4) | 2173(66.9) | 1 274(63.3) | 44(69.8) |  |  |
| Master degree | 2 216(20.5) | 860(16.3) | 804(29.4) | 552(19.7) |  |  | 772(22.5) | 42(7.4) | 1 402(20.6) |  |  | 1 231(22.4) | 525(16.2) | 452(22.5) | 8(12.7) |  |  |
| Doctor degree | 380(3.5) | 163(3.1) | 150(5.5) | 67(2.4) |  |  | 220(6.4) | 3(0.5) | 157(2.3) |  |  | 294(5.4) | 36(1.1) | 45(2.2) | 5(7.9) |  |  |
| **Proportion of the number of pharmacists to the total number of health**   **professionals**  **(The average of 143 sample health facilities was 4.8%)** | | | | | 1.2 | 0.87 |  |  |  | **24.3** | **<0.01** |  |  |  |  | **76.4** | **<0.01** |
| <5% | 97(67.8) | 43(65.2) | 25(73.5) | 29(67.4) |  |  | 36(84.1) | 0(0.0) | 61(65.2) |  |  | 54(83.1) | 1(3.3) | 24(92.3) | 18(81.8) |  |  |
| 5%-8% | 26(18.2) | 12(18.2) | 6(17.6) | 8(18.6) |  |  | 7(15.9) | 4(57.1) | 15(16.3) |  |  | 5(7.7) | 15(50.0) | 2(7.7) | 4(18.2) |  |  |
| ＞8% | 20(14.0) | 11(16.7) | 3(8.8) | 6(14.0) |  |  | 0(0.0) | 3(42.9) | 17(18.4) |  |  | 6(9.2) | 14(46.7) | 0(0.0) | 0(0.0) |  |  |
| **Number of sample hospitals** | **143** | 66 | 34 | 43 |  |  | 43 | 7 | 93 |  |  | 65 | 30 | 26 | 22 |  |  |
